# Hexane extract from black soldier fly prepupae: A novel immunomodulatory strategy against *Aeromonas hydrophila* infection in zebrafish

**DOI:** 10.14202/vetworld.2024.1655-1660

**Published:** 2024-07-30

**Authors:** Dahliatul Qosimah, Indah Amalia Amri, Dyah Ayu Oktavianie A. Pratama, Fajar Shodiq Permata, Noorhamdani Noorhamdani, Dhelya Widasmara, Jasni Sabri

**Affiliations:** 1 Laboratory of Veterinary Microbiology and Immunology, Faculty of Veterinary Medicine, Universitas Brawijaya, Malang, East Java 65151, Indonesia; 2 Laboratory of Veterinary Anatomical Pathology, Faculty of Veterinary Medicine, Universitas Brawijaya, Malang, East Java 65151, Indonesia; 3 Laboratory of Veterinary Anatomy and Histology, Faculty of Veterinary Medicine, Universitas Brawijaya, Malang, East Java 65145, Indonesia; 4 Department of Medical Microbiology, Faculty of Medicine, Universitas Brawijaya, Malang, East Java 65145, Indonesia; 5 Department of Dermatology and Venereology, Faculty of Medicine, Universitas Brawijaya, Dr. Saiful Anwar Regional Hospital, Jl. Jaksa Agung Suprapto 2, Malang, East Java 65111, Indonesia; 6 Faculty of Veterinary Medicine, Universitas Brawijaya, Malang, East Java 65151, Indonesia

**Keywords:** *Aeromonas hydrophilia*, black soldier fly larvae, hexane extract, immune modulation, zebrafish

## Abstract

**Background and Aim:**

*Aeromonas hydrophila* infections in fish result in significant financial losses within aquaculture. Previous research indicates black soldier fly (BSF) prepupae provide immunomodulatory benefits through their fatty acids, chitin, and proteins. The study evaluated the impact of hexane extract from black soldier fly prepupae (HEBP) on interleukin (IL)-4 and IL-10 cytokine expression in zebrafish, both infected and uninfected with *A. hydrophila*.

**Materials and Methods:**

Adult zebrafish (aged 4–5 months) was assigned to a negative control group (fed commercial feed), a positive control group (commercial feed + *A. hydrophila* infection at 10^7^ colony-forming unit/mL), and three treatment groups (T1, T2, T3) that received HEBP at doses of 1000; 2000 and 4000 mg/kg feed for 30 days, respectively. *A. hydrophila* infection was introduced on day 31 through immersion. Analysis of IL-4 and IL-10 expression in the head kidney trunk region (body without head and tail) through quantitative polymerase chain reaction was conducted on day 33.

**Results:**

The HEBP modulated the immune response to *A. hydrophila* infection at a concentration of 1000 mg/kg feed, as evidenced by an increase in IL-4 and IL-10 expression in the groups not infected with the bacteria. However, these cytokines were decreased in the infected groups.

**Conclusion:**

A feed concentration of 1000 mg/kg HEBP was identified as optimal for cytokine modulation. This discovery marks a significant advancement in the development and benefit of a natural extract-based immunomodulator in a zebrafish model, which is potentially immunotherapeutic against bacterial infections in fish for the aquaculture industry.

## Introduction

*Aeromonas hydrophila* can cause severe diseases in fish populations in aquaculture, resulting in mass fatalities [1]. The substantial economic losses include treatment costs, replacement of deceased fish, decreased production, and reputational damage to fish farmers [2]. The bacterium quickly spreads and easily transmits among vulnerable fish populations in aquatic environments. Effective management of *A. hydrophila* infections is essential for the health and productivity of fish in the aquaculture industry [3]. Although efforts to control these infections have been implemented, such as using antibiotics and vaccination, several challenges remain. Long-term antibiotic use can lead to bacterial resistance and increased environmental contamination risk [4]. Vaccination faces limitations, particularly due to its high cost and complex manufacturing processes that affect its effectiveness [5]. Adopting a more holistic and sustainable approach to management is essential. Addressing *A. hydrophila* infections is crucial to decrease infection rates, minimize economic losses, and maintain a healthy fish population. The combat of *A. hydrophila* infection can be approached through increasing probiotics usage [6], improving aquaculture practices [7], and deploying immunomodulatory agents [3, 8].

Black soldier fly larvae (BSFL)/prepupae offer various benefits for animal health, including immunomodulatory [9] and antimicrobial properties [10]. BSFL serve as immunomodulators by boosting the macrophage’s response to infections and lessening inflammation [11–13]. On the other hand, BSFL can also produce natural antimicrobial compounds that help combat a range of microbes [14] and decrease oxidative stress [15], potentially improving animal health. BSFLs boast a wealth of nutrients, including protein, healthy fats, and vitamins, that contribute to improved physical and overall health [16]. Due to its potential for enhancing animal health and welfare, research into using BSFL in animal feed has gained significant attention. Interleukin (IL)-4 promotes Th2 cell differentiation and subsequently aids in bacterial infections’ control [17, 18]. IL-10 is an anti-inflammatory cytokine that can inhibit excessive immune responses and prevent tissue damage caused by over-inflammation due to bacterial infections [19, 20]. BSFL encompass fats and essential fatty acids, such as omega-3 and omega-6, which control immune responses [16, 21].

The production of IL-4 and IL-10 in zebrafish infected with *A. hydrophila* by hexane extract from black soldier fly prepupae (HEBP) remains unclear. The study assessed the impact of HEBP on IL-4 and IL-10 production in zebrafish infected with *A. hydrophila*. Based on a clearer comprehension of HEBP’s role in immune responses in zebrafish, this study sought to provide new insights into bacterial infection control strategies in aquaculture, focusing on *A. hydrophila*.

## Materials and Methods

### Ethical approval

This study has received ethics approval from the Ethics Committee of Universitas Brawijaya (Approval No. 035-KEP-UB-2022).

### Study period and location

This study was conducted from June to September 2023 at the Laboratory of Fish Parasites and Diseases, Faculty of Fisheries and Marine Sciences, Universitas Brawijaya.

### Zebrafish

Adult zebrafish aged 4–5 months were obtained from the reproduction laboratory at the Faculty of Fisheries and Marine Sciences, Universitas Brawijaya, Malang, Indonesia. The selected zebrafish were from healthy breeders with no history of illness living in the maintenance pond.

### Preparation of HEBP

The fermentation medium comprised 150 g of tofu dregs and 150 g of mixed fruits (including papaya, guava, starfruit, avocado, watermelon, apple, and pear), with each fruit equally proportioned. This mixture was subsequently fermented using a blend of effective microorganisms 4, molasses, water, and the medium at ratios of 2:1:50:500 mL, respectively. Fermentation was performed for 48 h. BSFL sourced from a maggot farm in Lamongan, East Java, Indonesia, were introduced into this medium and allowed to grow until day 25. The prepupae were extracted, euthanized using hot water, washed, and dehydrated in a microwave (Sharp, Japan) until they reached a dry, crispy texture [22]. The dried BSFL was then ground into a powder. One hundred grams of this powder were placed in a dark bottle and mixed with 1 L of n-hexane for maceration over a period of 24 h. The macerate was filtered, and the residue was macerated up to 3 times or until the filtrate color became nearly clear. The collected filtrate was then concentrated using a rotary evaporator at 40°C–60°C [23], producing 2 g of concentrated extract.

### Zebrafish maintenance and bacterial induction

Our study consisted of 8 treatment groups. Adult zebrafish were randomly allocated to aquariums maintained at 25°C and pH 6.8 ± 0.46. Each treatment group utilized 90 fish, divided into three replicates. Each replicate comprised three 15-L aquariums, with 30 fish per aquarium. In total, each treatment group had nine aquariums. The sample size was determined as described by Qosimah *et al*. [24]. Before introducing the fish, the aquariums were cleaned with disinfectants (chlorine and sodium thiosulfate) and aerated for 24 h. The fish were acclimatized for 7 days with commercial basal feed of MS Prima Feed PF 500 (PT Multi Sukses Sejahtera, Indonesia) under environmental conditions to stabilize their physical state before treatment.

In this study, HEBP supplementation and *A. hydrophila* infection were independent variables, whereas increased IL-4 and IL-10 expressions are expected outcomes (alternative hypothesis: H1). The study comprised eight treatment groups: A negative control group without infection (baseline), received commercial feed without HEBP and infection; a negative control group with infection, received commercial feed (without HEBP) and infected with *A. hydrophila*; treatment groups without infection, received commercial feed supplemented with HEBP but without *A. hydrophila* infection (T1, T2, and T3 at doses of 1000, 2000, and 4000 mg/kg feed, respectively); and treatment groups with infection received commercial feed supplemented with HEBP and infected with *A. hydrophila* (T1 infection, T2 infection, and T3 infection groups at doses of 1000, 2000, and 4000 mg/kg feed). The doses for the groups were decided based on the study of Fang *et al*. [25]. The treatments were administered for 30 days. On day 31, the fish were infected by immersion in an inoculum containing *A. hydrophila* at a concentration of 10^7^ colony-forming unit (CFU)/mL. The inoculum was prepared by suspending one colony of bacteria in 10 mL of brain heart infusion broth (BHIB) medium, adjusting the concentration to match a 0.5 McFarland standard (approximately 10^8^ CFU/mL), and then diluting it to 10^7^ CFU/mL.

### Gene expression measurements using quantitative polymerase chain reaction

Gene expression was analyzed using head and kidney extracts with a Bio-Rad CFX Maestro 2.2 (Bio-Rad, USA) real-time polymerase chain reaction (PCR) system. A 10 μL PCR reaction containing EvaGreen Supermix, (Bio-Rad) primers, ddH_2_O, and complementary DNA (cDNA). The computational determination of threshold cycles (Ct) facilitated the calculation of relative messenger RNA (mRNA) levels for each gene. β-Actin served as a calibrator to assess cDNA quality and normalize expression levels. The expression fold change was calculated as 2^-ΔCt^ based on ΔΔCt values. The quantitative PCR (qPCR) protocol for β-actin, IL-4, and IL-10 included multiple temperature cycles across 45 cycles, and data analysis was performed using CFX Manager 2.0 software (Bio-Rad). Table-1 presents the qPCR primer design for the zebrafish used in the study. Beta-actin served as the internal control gene, normalizing target gene expression levels to account for variations in mRNA levels and sample processing [26]. This normalization ensured that any observed differences in gene expression were attributed solely to changes in the genes of interest. Harvesting was conducted in a cold environment using ice stones for anesthesia, minimizing stress to ensure sample integrity.

**Table-1:** qPCR primer design for zebrafish (Private documentation).

Gene	Sequence	Product length (bp)	Input PCR template
Beta-actin	Forward primers: GTATGGCGCATTGACTCAGG Reverse primer: AAGCTCTCCCCTGTTAGACAA	67	*Dano rerio* beta-actin mRNA, complete CDS; AF057040.1
IL-10	Forward primer: GACCATTCTGCCAACAGCTC Reverse primer: ACCATATCCCGCTTGAGTTCC	102	*Danio rerio* IL-10 mRNA: NM_001020785.2
IL-4	Forward primer: AACTCTCTGCCAAGCAGGAA Reverse primer: CTACCGTGCATTCCCCCGAG	137	*Danio rerio* IL-4 (NM_001170740.1) mRNA

mRNA=Messenger RNA, qPCR=Quantitative polymerase chain reaction, IL: Interleukin

### Statistical analysis

The one-way analysis of variance was used to quantitatively analyze the gene expression data derived from qPCR tests at a 5% confidence level. *Post hoc* multiple comparisons were made using Tukey’s method with GraphPad Prism software version 8.4.0 (GraphPad, USA).

## Results

**Figure-1:**
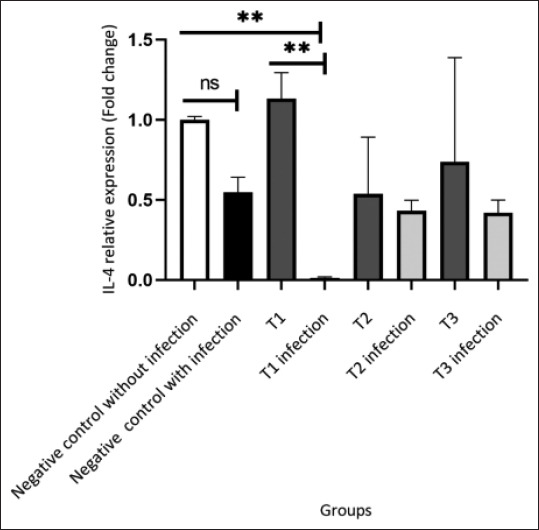
In the T1 group, IL-4 expression was increased relative to the negative control group, which was not infected. Conversely, IL-4 expression was decreased in groups infected with *Aeromonas hydrophila*, although this reduction was not statistically significant (p > 0.05). The increase in IL-4 expression was greater in the T1 group than in the other groups, with the only significant difference observed between the T1 and T1 infection groups. The symbol “ns” indicates a non-significant difference (p > 0.05), and ** denotes a highly significant difference (p < 0.01). IL=Interleukin.

The study revealed that HEBP administration significantly affected IL-4 expression in both the *A. hydrophila*-infected and uninfected groups (p = 0.004). The negative control group without infection expressed basal levels of IL-4. In contrast, the negative control group with infection exhibited a decrease in IL-4 expression, indicating activation of the immune response due to infection. The relative expression of IL-4 in the negative control without infection was not significantly different from that in the negative control with infection (p > 0.05). However, the average negative control without infection (1.00 ± 0.02) was higher than the negative control with infection (0.55 ± 0.10). IL-4 gene expression in the treatment groups without infection, T1 (1.13 ± 0.16), T2 (0.54 ± 0.36), and T3 (0.74 ± 0.65) did not significantly differ from the infected groups (p > 0.05) for T1 infection (0.01 ± 0.01), T2 infection (0.43 ± 0.06), and T3 infection (0.42 ± 0.08), although on average, the treatment groups without infection showed higher IL-4 expression compared to the infected ones. On average, the highest IL-4 expression was observed in the T1 group at a feed concentration of 1000 mg/kg compared with all other treatment and negative control groups (Figure-1).

**Figure-2:**
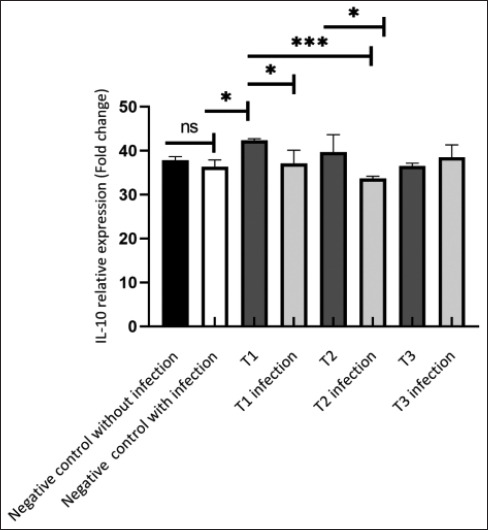
HEBP on IL-10 Expression. The T1 group exhibited a significant increase in IL-10 expression compared with the negative control group (p < 0.05). In addition, this group demonstrated a highly significant increase in IL-10 expression relative to both the T1 infection group and the T2 infection group (p < 0.001). In this notation, “ns” represents a non-significant difference; * indicates a significant difference (p < 0.05); and ** denotes a highly significant difference (p < 0.001). HEBP=Hexane extract from black soldier fly prepupae, IL=Interleukin.

The administration of HEBP significantly influenced IL-10 expression in both the treatment groups without infection and the treatment groups with infection (p = 0.0006). IL-10 expression in the negative control group without infection (37.83 ± 0.84) showed an increase compared to a negative control group with infection (36.3 ± 1.58), although not significantly (p > 0.05) (Figure-2).

The T1 treatment group, which was not infected with bacteria at a concentration of 1000 mg/kg of feed, exhibited the highest IL-10 expression compared with the other treatments, although the difference was not significant. The administration of HEBP to the T1 and T2 groups, which were not infected with *A. hydrophila*, showed higher IL-10 expressions, with T1 at 42.29 ± 0.44, T2 at 39.65 ± 4.02, and T3 at 36.52 ± 0.63, compared with those infected with bacteria, which were T1 infection at 37.10 ± 2.99, T2 infection at 33.64 ± 0.56, and T3 infection at 38.47 ± 2.85. The increase was not statistically significant. The T1 group exhibited higher IL-10 expression than the T2 and T3.

## Discussion

HEBP administration significantly influenced IL-4 and IL-10 expression in *A. hydrophila*-infected zebrafish. In the negative control group with infection, the expression levels of IL-10 and IL-4 were lower than those in the negative control group without infection and with no bacteria. During bacterial infection, the body responds by producing more proinflammatory cytokines to combat pathogens [27], but bacteria can evade this response and multiply within the fish body. During bacterial infection, the body produces more proinflammatory cytokines to eliminate pathogens [27], allowing bacteria to circumvent immune response regulation and multiply within the fish body.

The study indicated that zebrafish infected with *A. hydrophila* showed increased IL-10 and IL-4 expressions in the early stages of infection to alleviate inflammation, decreased in subsequent stages after successfully mitigating the bacteria, and later increased again to maintain body homeostasis. IL-10 and IL-4 regulate the immune response to balance inflammatory and anti-inflammatory responses [28]. IL-10 suppresses the secretion of tumor necrosis factor-alpha and IL-1β [29], thus reducing the risk of hyperinflammation and undesirable tissue injury [28]. B cells undergo proliferation and differentiation for antibody production on exposure to IL-4 [30]. IL-4 instigated the Th2 cell immune response by generating mucus and altering macrophage differentiation, thereby protecting fish from excessive inflammation [31].

An increase in IL-4 expression at a 1000 mg/kg feed concentration was optimal for inducing immunity, while decreases were observed at 2000 mg/kg and increases resumed at 4000 mg/kg. The biological activity of bioactive compounds in the extract increased at lower concentrations. BSFL contains bioactive compounds such as proteins, fats, fatty acids, and amino acids, which have potential as immunomodulators [32, 33]. Reducing the amount of HEBP in the feed could increase these compounds, thereby stimulating a higher IL-4 response from immune system. A 1000 mg/kg feed concentration can promote increased IL-4 production in fish by means of the balanced bioactive compounds. Further investigation is needed to unravel the specific processes underlying this intricate response pattern.

IL-10 expression peaked at 1000 mg/kg HEBP but decreased at 2000 and 4000 mg/kg. A decrease in IL-10 levels reveals the intricate interplay of different immune-modulating agents within HEBP, implying a weaker impact on the immune system. The intricate interplay among HEBP’s bioactive compounds impairs its effectiveness in stimulating the immune system [34, 35]. The observed decrease in immune response may be due to other factors, such as toxicity or antagonistic effects of certain compounds at higher concentrations [36, 37]. IL-10, an immune system inhibitor, curbs excessive inflammation response by decreasing pro-inflammatory cytokine generation and dampening immune cell activation. IL-10 is essential for balancing the immune response toward pathogens and commensal microbes [28]. This equilibrium prevents excessive defense mechanisms from damaging host tissue and ensures a healthy symbiosis.

This study identified tripeptide-3 and L-carnitine as the active constituents of HEBP (unpublished data). Tetrapeptide-3 regulates the immune system, preventing overreactions. Enhancing the proliferation and differentiation of specific cells can promote tissue healing and regeneration. This peptide can inhibit the activity of enzymes involved in the inflammatory process by reducing vascular permeability and alleviating the immune response [38]. L-carnitine boosts the performance of immune cells like lymphocytes, macrophages, and NK cells, significantly enhancing their ability to combat infections and diseases. It enhances the secretion of anti-inflammatory cytokines and decreases oxidative stress, thus preventing cellular damage [39]. The gut microbiota can metabolize L-carnitine, which plays a significant role in immunity regulation and gut microbiota balance [40]. This study suggests HEBP’s potential in fish nutrition for use as a feed additive and in improving fish health management against infectious diseases by intensifying the immune responses of IL-4 and IL-10.

## Conclusion

This study demonstrated HEBP’s immunomodulatory effects in zebrafish infected with A. hydrophila, specifically through IL-4 and IL-10 regulation. While using crude BSF prepupae extracts limited identification of specific active compounds, our findings provide a foundation for future research. Further analysis to isolate and characterize these components could yield novel therapeutic options for bacterial infections in aquaculture. This zebrafish model contributes valuable insights to fish immunology and opens avenues for innovative disease management strategies.

## Authors’ Contributions

DQ: Conceptualized the study, prepared the outlines, conducted the study, contributed to the acquisition and interpretation of data and drafted and revised the manuscript.JS: Contributed to the analysis and interpretation of data and edited the manuscript. DAOAP, FSP, IAA, DW, and NN: Contributed equally to the acquisition and analysis of data and drafted and revised the manuscript. All authors have read, reviewed, and approved the final manuscript.
